# Evidence in duck for supporting alteration of incubation temperature may have influence on methylation of genomic DNA

**DOI:** 10.3382/ps/pev201

**Published:** 2015-09-09

**Authors:** Xi-ping Yan, He-he Liu, Jun-ying Liu, Rong-ping Zhang, Guo-song Wang, Qing-qing Li, Ding-min-cheng Wang, Liang Li, Ji-wen Wang

**Affiliations:** Farm Animal Genetic Resources Exploration and Innovation Key Laboratory of Sichuan Province, Sichuan Agricultural University, Ya'an, Sichuan 625014, P.R. China

**Keywords:** duck, incubation temperature, DNA methylation, embryonic period

## Abstract

Incubation temperature has an immediate and long-term influence on the embryonic development in birds. DNA methylation as an important environment-induced mechanism could serve as a potential link between embryos’ phenotypic variability and temperature variation, which reprogrammed by DNA (cytosine-5)-methyltransferases (*DNMTS*) and Methyl-CpG binding domain proteins (*MBPS*) 3&5 (*MBD3&5*). Five genes in *DNMTS* and *MBPS* gene families were selected as target genes, given their important role in epigenetic modification. In this study, we aimed to test whether raising incubation temperature from 37.8°C to 38.8°C between embryonic days (ED) 1–10, ED10–20 and ED20–27 have effect on DNA methylation and whether *DNMTS*, *MBPS* play roles in thermal epigenetic regulation of early development in duck. Real-time quantitative PCR analysis showed that increased incubation temperature by 1°C has remarkably dynamic effect on gene expression levels of *DNMTS* and *MBPS*. Slight changes in incubation temperature significantly increased mRNA levels of target genes in breast muscle tissue during ED1–10, especially for *DNMT1*, *DNMT3A* and *MBD5*. In addition, higher temperature significantly increased enzyme activities of DNMT1 in leg muscle during ED10–20, liver tissue during ED1–10, ED20–27 and DNMT3A in leg muscle and breast muscle tissue during ED10–20. These results suggest that incubation temperature has an extended effect on gene expression levels and enzyme activities of *DNMTS* and *MBPS*, which provides evidence that incubation temperature may influence DNA methylation in duck during early developmental stages. Our data indicated that *DNMTS* and *MBPS* may involved in thermal epigenetice regulation of embryos during the early development in duck. The potential links between embryonic temperature and epigenetic modification need further investigation

## INTRODUCTION

The complete description of developmental series of avian embryos can be traced back to the end of the 19th century (Duval, 1889; Keibel, 1900). What's more, Hamburger and Hamilton estabilished a system of normal 42 stages of embryonic development available for birds, which represent a general developmental pattern (Hamburger and Hamilton, 1951). Numerous studies have well documented the detailed processes of early growth and development of the embryos based on morphological characters in avian (Romanoff, 1960; Starck and Ricklefs, 1998; Bellairs and Osmond, 2005; Patten, 2008). Furthermore, the complex physiological processes of incubation were previously described (Olsen, 1942; Sturkie, 1965; Freeman and Vince, 1974; Whittow, 1999). All of these studies indicate that the incubation stages is important for embryonic development and organogenesis.

The embryonic stages is apt to be influenced by surrounding physical factors, which consequently could lead to changes in particular phenotypes and eventially may obtain environmental adaptability (Feil and Fraga, 2012; Jablonka, 2013; LaFreniere and MacDonald, 2013; Schlichting and Wund, 2014). Together, these environmental factors influence phenotypes in one generation, which could also affect phenotypes in subsequent generations through epigenetic mechanisms. In avians, incubation temperature as one of the most important environmental factors can affect embryonic development (Deeming, 2002; Oviedo-Rondón et al., 2008; Barri et al., 2011; Shim and Pesti, 2011), metabolism (Lourens et al., 2006; Willemsen et al., 2010) and even the development of birds during post-hatch stages (Piestun et al., 2009; Piestun et al., 2013). Similarly, a previous study reported that raising the incubation temperature by 1°C during ED5–8 results in an increase in semitendinosus muscle fibre amount at 16 days after hatching in turkeys (Maltby et al., 2004). In addition, Hammond et al. demonstrated that incubation temperature raised by 1°C for 3 days at early stages in chick, led to an increase in the growth rate of muscle and embryo activity (Hammond et al., 2007). Our initial data demonstrated that raising incubation temperature by 1°C during ED 11–24 has an inhibiting effect on immune function at the middle embryonic stages and has significant increased effects on lipid metabolism in ducklings during post-hatch stages (Liu et al., 2013; Wang et al., 2014). Therefore, it is fully confirmed that thermal manipulation during incubation stages has an extensive influence on embryonic development, adult phenotypes even their offspring phenotypes. (Piestun et al., 2008; Shinder et al., 2011; Renaudeau et al., 2012; Frésard et al., 2013)

Epigenetic modifications could be the potential link between the response of embryo phenotypic variability and certain environmental stimuli (Jaenisch and Bird, 2003). A previous study demonstrated that ducklings at slightly cooler incubation temperature experienced weaker immunity due to reduced response to new antigens; meanwhile, they predicted that thermal manipulation would induce phenotype variations that are characteristic of epigenetic modifications including variation in growth rate, body condition and immune response (DuRant et al., 2012).

DNA methylation is an essential epigenetic modification acquired in early life that stores epigenetic memory and therefore has an underlying effect on phenotypes of one generation or even the subsequent generations (Daxinger and Whitelaw, 2010). DNA (cytosine 5)-methyltransferases (*DNMTS*) and Methyl-CpG binding domain proteins *(MBPS*) are important components of the DNA methylation system, which involves the establishing and maintenance of DNA methylation patterns (Klose and Bird, 2006; Geiman and Muegge, 2010). Campos et al. reported that increasing embryonic temperature range in fish from 23–27°C to 23–31°C had a remarkable dynamic effect on gene expression of *DNMT3A*, which is a key gene regulating the de novo methylation process (Campos et al., 2012). In contrast, little is known about the effects of thermal stimuli on methylation of genomic DNA in birds.

Therefore, the aim of our present study was to test whether alteration of incubation temperature could affect DNA methylation and whether *DNMTS* and *MBPS* play roles in thermal epigenetic regulation of early development in duck. To conduct this, we artificially incubated duck eggs at different thermal treatment conditions, and then, *DNMTS* and *MBD3&5* genes expression levels and DNMTS enzyme activities were examined. These data prove the incubation temperature manipulation affect epigenetic modification of duck embryos and may provide clues for the research that related to the long-term temperature adaptability acquisition when birds exposed to the changing environmental temperature condition.

## MATERIALS AND METHODS

### Birds and Tissues

**Figure 1.**
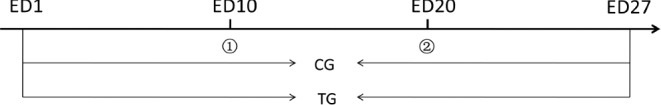
The basic treatment process. CG, TG mean control group and treatment group. Treatment was comprised of increasing the incubation temperature by 1°C during ED1–10, ED10–20, ED20–27. means at ED10, more than 15 qualified eggs were transferred from control group to treatment group and incubated at 38.8°C during ED10–20, while eggs in control group still incubated at 37.8°C. means at ED20, more than 15 qualified eggs were transferred from control group to treatment group and incubated at 38.8°C during ED20–27, while eggs in control group still incubated at 37.8°C. Samples were taken at ED10, 20 and 27.

A total of 100 eggs from breeders aged between 30 and 50 weeks. All eggs used in the study were obtained from Peking ducks (*Anas platyrhynchos domestica*). Peking duck eggs were selected from Sichuan Agricultural University Waterfowl Breeding Experiment Farm. Fresh eggs were artificially collected from nest boxes and storaged at the following environmental conditions no more than 7 days: room temperature of 15°C–18°C, relative humidity of 70–75%. These eggs were numbered and weighted individually at the range of 84.0 ± 2.0g and divided into two groups randomly. Eggs in the control group were transferred into the same incubation equipment controled by computer with fuzzy control system (Haijiang, Beijing, China) and maintained at a temperature of 37.8°C and relative humidity of 60–65% from ED1 to ED27; Meanwhile, eggs in the treatment group were incubated at 38.8°C, whereas other conditions remained identical with the control group. These eggs were turned once per hour during ED1–27. At ED10, all eggs were quality tested by candling to identify infertile eggs and dead embryos, then, unqualified eggs were removed from the subsequent experiment. The treatment group with 15 qualified eggs incubated at 38.8°C and the other conditions remained the same as the control group, during ED10–20. At ED20, 15qualified eggs were transferred from control group to treatment group and incubated at 38.8°C until the end of incubation. The incubation temperature design are listed in Figure 1. We divided the incubation stages into three phases, early embryonic stages (ED1–10), middle embryonic stages (ED10–20), later embryonic stages (ED20–27), which based on previous studies in birds (Collin et al., 2007; Hammond et al., 2007; Al-Musawi et al., 2012). Six ducklings were slaughtered by rapid bloodletting at ED10, 20 and 27. The leg muscle tissue, breast muscle tissue, heart tissue and liver tissue were isolated and frozen in liquid nitrogen for RNA extraction.

### RNA Isolation and Real-Time Quantitative PCR

Total RNA was extracted from the leg muscle tissue, breast muscle tissue, heart tissue and liver tissue by using Trizol reagent (Invitrogen, CA, USA) following the instructions of the manufacturer. Electrophoresis on 1% (w/v) agarose gel was used to assess RNA quality. For real-time PCR analysis, the total RNA from all samples was treated with DNase I for 10 min and then quantified by spectrophotometric absorbance at 260 nm and 280 nm. Absorbance ratios were greater than 1.9, which indicated high purity RNA. For genes unrecorded in the NCBI database, RT-PCR was used to obtain their sequences. The detailed information of RT-PCR primers and the real-time PCR primers are listed respectively in Table 1 and Table 2. Primers were designed using the primer 5 software (Primer Biosoft International, USA) and were synthesised by BGI (Beijing, China). 96-well iCyclerIQ5 (USA) and TaKaRa Ex Taq RT-PCR kit (Takara, Dalian, China) were used to measure the relative mRNA expression of genes. The real-time PCR reaction contained 1 μL of cDNA template, 12.5 μL of SYBR Premix Ex Taq, 10.5 μL of sterile water, and 0.5 μL of primer. Thermal cycling parameters were one cycle of initial denaturation at 95°C for 30 s, 40 cycles of 95°C for 10 s and 60°C for 40 s. The reactions of each sample were repeated three times. The relative mRNA expression levels of *DNMTS* and *MBD3&5* were calculated by the “normalized relative quantification’’ method followed by 2^−ΔΔCT^ (Livak and Schmittgen, 2001).

**Table 1.** Primers for PCR analysis

Gene		Primer sequence (5′-3′)	Product length(bp)	Tm(°C)
dnmt1	F	5′-GCTATGTCGCCCTGGATTTC-3′	507	64.1
	R	5′-CACAGGACTCCATACCCAAGAA-3′		
dnmt3a	F	5′-GGAGCACCCTTTGTTTATCG-3′	481	56.8
	R	5′-TTCGGAGGCAATGTAGCG-3′		
dnmt3b	F	5′-CCACTACACCGACGTTTCCA-3′	443	60.3
	R	5′-GCCTCCACCACTTTCTCCTC-3′		
mbd3	F	5′-TGGACCTCAGCACTTTCGAC-3′	387	61.6
	R	5′-GCAGAGCACTAGCGATAGCA-3′		
mbd5	F	5′-GCTATGTCGCCCTGGATTTC-3′	525	63.5
	R	5′-CACAGGACTCCATACCCAAGAA-3′		

*Notes*: F, forward primer; R, reverse primer.

**Table 2.** Primers for real-time PCR

Gene		Primer sequence (5′-3′)	Product length(bp)	Tm(°C)
β**-**actin	F	5′-GCTATGTCGCCCTGGATTTC-3′	168	60
	R	5′-CACAGGACTCCATACCCAAGAA-3′		
GADPH	F	5′-AAGGCTGAGAATGGGAAAC-3′	254	60
	R	5′-TTCAGGGACTTGTCATACTTC-3′		
18S rRNA	F	5′-TTGGTGGAGCGATTTGTC-3′	129	60
	R	5′-ATCTCGGGTGGCTGAACG-3′		
dnmt1	F	5′-GAAATCGACGGTCGTCTCCTC-3′	149	60
	R	5′-TCAGCAACGGCAAGCCTAAC-3′		
dnmt3a	F	5′-GAGGCAATGTAGCGATCCACC-3′	160	60
	R	5′-CCAACAACCACGACCAGGAGT-3′		
dnmt3b	F	5′-ACAGGCAAAGTAATCCTTGAGCG-3′	110	60
	R	5′-CCGACGTTTCCAACATCAGC-3′		
mbd3	F	5′-ACCTTATTGCTGGGATGGTTTGT-3′	175	60
	R	5′-GCACGGGAAAGATGTTGATGAG-3′		
mbd5	F	5′-GTTTCCATAGCCACCTCCTCC-3′	120	60
	R	5′-TGTTTCTGCCATTGACACCACT-3′		

*Notes*: F, forward primer; R, reverse primer.

### Elisa

**Figure 2.**
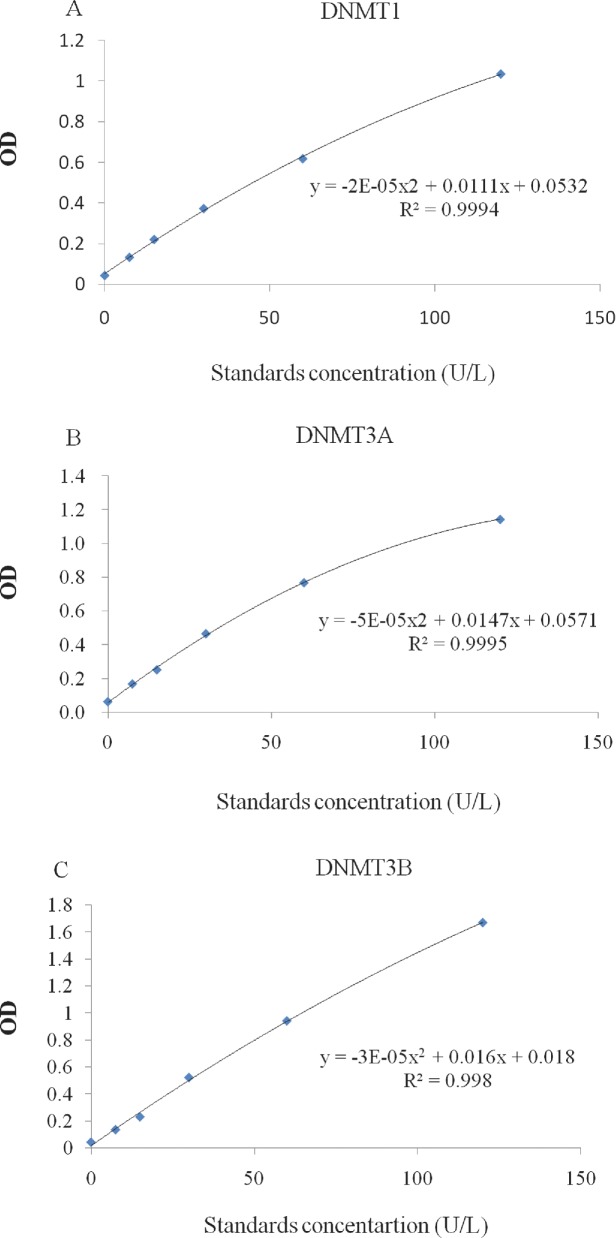
Standard curve is used to determine DNMTS concentration in each sample. A. Standard curve of enzyme DNMT1 activity, B. Standard curve of enzyme DNMT3A activity, C. Standard curve of enzyme DNMT3B activity.

DNMTS concentration of samples was measured by using DNMTS ELISA Kits (Jijin, Shanghai, China) following the manufacturer's instructions. The standard curve was generated by standard samples used for determining the concentration of unknown samples. All standard curves had r^2^ values > 0.9900 (Figure 2).

### Statistical Analysis

All data were subjected to M.S. Excel program and performed in SAS V.8.0 (SAS Institute Inc., Cary, NC, USA). All data were listed as the format of mean±S.D. For the gene mRNA analyses, n = 6; For the enzyme activities measurement, n = 6. One-way analysis of variance was used to determine the statistical significance between the groups. The significance of data was recognised at level of *P* < 0.05.

## RESULTS

### Gene Expression Levels of DNMTS and MBD3&5

**Figure 3.**
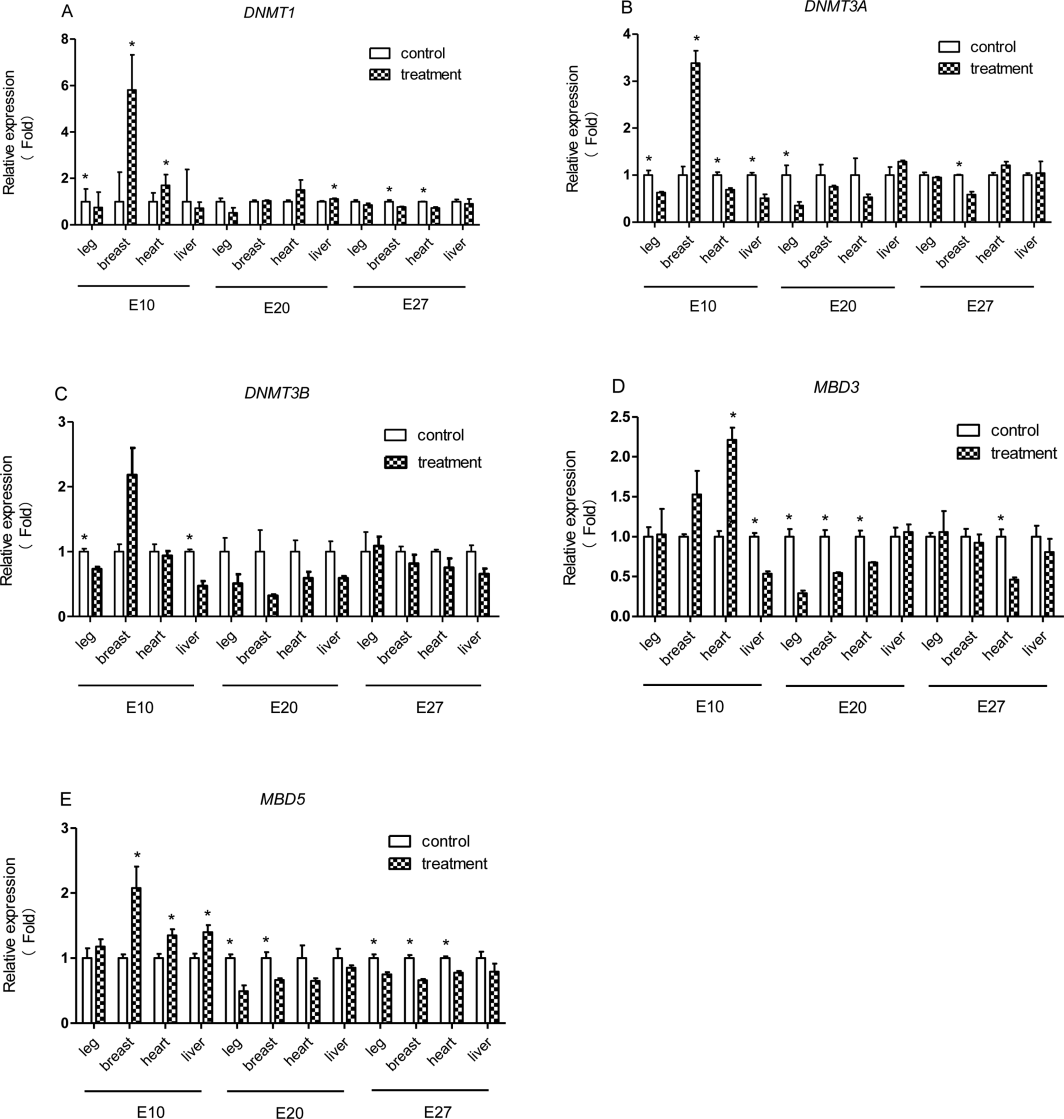
The relative mRNA expression levels of *DNMTS* and *MBD3&5* in treatment and control groups (n = 6) were confirmed by real-time RCR. A. Relative gene expression of *DNMT1*, B. Relative gene expression of *DNMT3A*, C. Relative gene expression of *DNMT3B*, D. Relative gene expression of *MBD3*, E. Relative gene expression of *MBD5*. Label * on the bar means a significant difference at the level *P* < 0.05.

To investigate the response to temperature manipulation during embryonic periods, gene expression levels of *DNMTS* and *MBD3&5* were detected by using real-time PCR in leg muscle tissue, breast muscle tissue, heart tissue and liver tissue. Data are listed in Figure 3. These results showed significantly stage-specific responses.

Relative expression patterns were markedly dynamic in *DNMTS* during the entire embryonic phases; during the early embryonic stages we found gene expression levels of *DNMTS* in leg muscle tissue was significantly lower under thermal treatment conditions (*P* < 0.05) (Figure 3). Gene expression level of *DNMT1* and *DNMT3A* in breast muscle tissue were up-regulated in the treatment group with significant change (*P* < 0.05) (Figure 3A and B). Additionally, mRNA expression levels of *DNMT1* in heart tissue were up-regulated in the treatment group and exhibited a significant difference (*P* < 0.05) (Figure 3A), however, *DNMT3A* showed an opposite gene expression trend with significant change (*P* < 0.05) (Figure 3B). The gene expression levels of *DNMT3A* and *DNMT3B* in liver tissue were significantly lower under thermal treatment conditions (*P* < 0.05) (Figure 3B and C); During the middle embryonic stages, the mRNA levels of *DNMT1* in liver tissue was significantly increased in the treatment group (*P* < 0.05), and mRNA levels of *DNMT3A* in leg muscle tissue significantly decreased in the treatment group (*P* < 0.05) (Figure 3A and B). During the later embryonic stages, gene expressions levels of *DNMT1*, *DNMT3A* in breast muscle tissue and *DNMT1* in heart tissue was significantly down-regulated in the treatment group (*P* < 0.05) (Figure 3A and B).

Gene expression trends of *MBD3&5* were similar during the developmental stages, especially during the middle and later period, with *MBD3&5* having higher gene expression levels in heart tissue and *MBD5* in liver tissue in the treatment group with significant changes during the early embryonic stages (*P* < 0.05) (Figure 3D and E). During the middle embryonic stages, gene expression levels of *MBD3* in leg muscle tissue (Figure 3D), heart tissue, *MBD5* in leg muscle tissue, breast muscle tissue (Figure 3E) was down-regulated in the treatment group and exhibited a significant difference (*P* < 0.05). Additionally, gene expression levels of *MBD3* in heart tissue (Figure 3D), and *MBD5* in leg muscle tissue, breast muscle tissue, heart tissue (Figure 3E) were significantly down-regulated under thermal treatment conditions during the later embryonic stages (*P* < 0.05).

### Enzyme Activities of DNMTS

**Figure 4.**
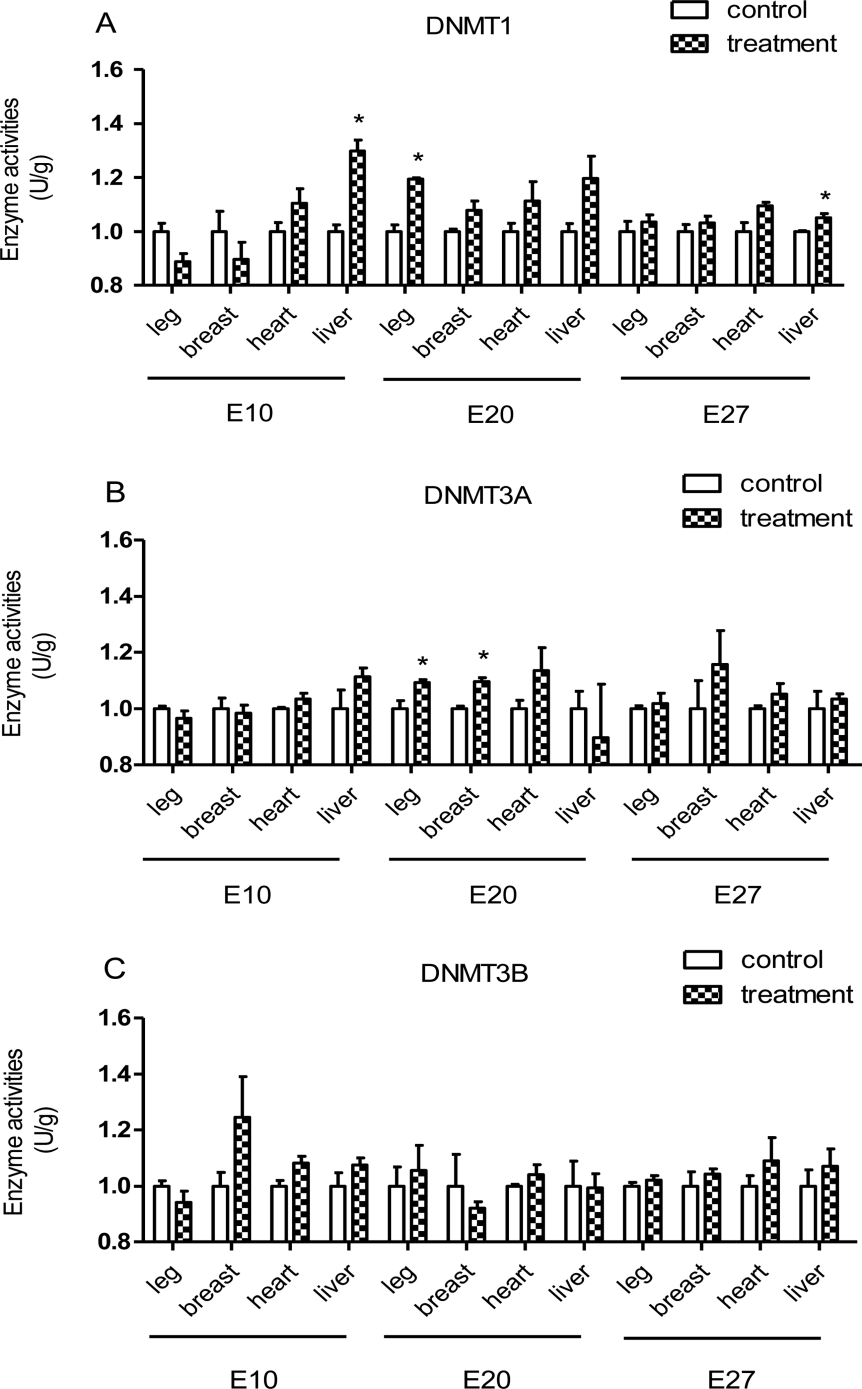
The enzyme activities of DNMTS in treatment and control groups (n = 6) were confirmed by DNMTS ELISA Kits. A. Determination of DNMT1 enzyme activity, B. Determination of DNMT3A enzyme activity, C. Determination of DNMT3B enzyme activity. Label * on the bar means a significant difference at the level *P* < 0.05.

To further test the relationship between incubation temperature and DNA methylation, enzyme activities of DNMTS were measured in the current study. The ELISA data in Figure 4 showed that the enzymatic activity of DNMT1 in liver tissue during the middle and later embryonic stages and of DNMT1 in leg muscle tissue (Figure 4A) during the middle embryonic stages were significantly enhanced by thermal manipulation (*P* < 0.05). The enzyme activities of DNMT3A (Figure 4B) during the middle embryonic stages show significant difference between the two groups in leg muscle tissue and breast muscle tissue (*P* < 0.05). For the other tissues during the entire embryonic phases measured, few significant differences were found.

## DISCUSSION

Incubation period is very important for embryonic development and organogenesis, and related studies have been conducted in avian to signify the importance of incubation stages. (Yılmaz et al., 2011; Al-Musawi et al., 2012; Schwabl et al., 2012; Zhang et al., 2012; Chen et al., 2013; Kim et al., 2013; Vince, 2013). Previous studies in Montastraea faveolata (Voolstra et al., 2009) and Atlantic cod embryos (Skjaerven et al., 2014) showed that increasing incubation temperature during the embryonic stages could influence gene expression. In the current studies, *DNMTS* and *MBD3&5* gene expressions changed significantly in different tissues at embryonic stages. Our results are consistent with these studies and provide available evidence that embryonic stage is crucial for gene expression.

Environmental factors, i.e., temperature (Nichelmann, 2004), chemicals (Vandegehuchte et al., 2010), or nutrition (Anderson et al., 2012) affect phenotypes during early developmental stages. The changes of DNA methylation may prove that environmental factors could cause the phenotypic variability. Change in incubation temperature is one of the most significant stressors for bird, so the current study was conducted to determine whether altering incubation temperature could influence methylation of genomic DNA. DNA methylation during embryonic stages is a dynamic mechanism, which plays a crucial role in vertebrate development. DNMTS are responsible for regulating DNA methylation in the genome and they are essential for this process (Kamei et al., 2010). In mammals, the *DNMTS* gene family consists of *DNMT1*, *DNMT3A*, and *DNMT3B*, which are grouped by the differences of structure and function (Goll and Bestor, 2005). DNMTS have been confirmed have catalytic activity in vivo (La Salle and Trasler, 2006). It has been reported that DNMT1 is regarded as the major maintenance methyltransferase, whereas, DNMT3A and DNMT3B have novo methylation activity and act as important methylation repair enzymes (Chen et al., 2003). On the other hand, MBD3&5 are members of the Methyl-CpG binding domain protein (MBPS) family, which have crucial roles in transcriptional regulation and development (Fournier et al., 2012). These genes were selected in the current study due to their critical role in epigenetic modification. We first examined gene expressions since previous studies have reported that a single physical factor like temperature can influence gene expression levels. We show here that embryos studied after thermal manipulation have stage-specific changes in mRNA levels for target genes; meanwhile, mRNA expression profiles of *DNMTS* and *MBD3&5* were highly dynamic during the embryonic development, showing their essential function during the early developmental stages. For the *DNMTS* gene family, gene expression levels in leg muscle tissue and liver tissue were significantly down-regulated under thermal manipulation conditions during ED1–10 (*P* < 0.05) except *DNMT1*. We also found that *DNMTS* gene expression levels in breast muscle tissue were up-regulated by a higher incubation temperature during ED1–10. This effect showed that the expression profile of the de novo and maintenance methyltransferases is tissue-specific during embryonic developmental stages (Li et al., 1992). Gene expressions levels of *MBD3&5* were found to be more dynamic than *DNMTS*. Relative expression patterns of *MBD3&5* were similar in different tissues during all embryonic stage investigated, but significant differences between incubation temperatures were observed during ED20–27. The results showed that the gene expression levels of *MBD3&5* in leg muscle tissue, breast muscle tissue, heart tissue were down-regulated (*P* < 0.05) in the treatment group. While during ED1–10, gene expression levels of *MBD3* in heart tissue and *MBD5* in breast muscle tissue, heart tissue, liver tissue were significantly up-regulated (*P* < 0.05) under thermal manipulation conditions, the opposite expression trend was observed in liver tissue. It seems that higher incubation temperature represses gene expression, particularly for *MBD3&5* at later embryonic stages. For the *DNMTS* gene family, the treatment incubation temperature has a complex effect on expression levels during all embryonic stages, especially in the early developmental period, except for *DNMT3B*. The different gene expressions of dnmts and *MBD3&5* levels under slightly higher embryonic incubating temperature suggest that they may have different roles in epigenetic gene regulation during embryonic development. In order to further validate our hypothesis that DNA methylation indeed can be influenced by temperature, enzyme activities of DNMTS were measured in the current study and significant differences were observed in the enzyme activity of DNMT1 in liver tissue during ED1–10, ED20–27, in leg muscle tissue during ED10–20. Enzyme activity of DNMT3A in leg muscle tissue and breast muscle tissue were significantly enhanced under thermal manipulation conditions. We show here that embryos researched after higher incubation temperature treatment also have a stage-specific increase in enzyme activities for DNMTS, both de novo and the maintenance methyltransferases that play an important role in embryonic development. We also demonstrate that higher thermal manipulation has an increased effect on enzyme activities of DNMTS. Previous work reported in fish that *DNMT3A* gene expressions increased during all developmental stages when incubation temperature was increased moderately (Campos et al., 2012). These results suggest that animals that are sensitive to incubation temperature may have similar epigenetic modifications related to DNA methylation. Collectively, our findings in duck indicate that incubation temperature stimuli have an extended effect on DNA methylation, which may provide evidence that links epigenetic modification to thermal manipulation during embryonic periods. Our findings may support the hypothesis that *DNMTS* and *MBPS* play roles in thermal epigenetic regulation of the early development in duck. To our present knowledge, this is the first evidence proving that tissue-specific and stage-specific DNA methylation is caused by environmental stimuli in avian species. However, more work is required in the future to ascertain if these expression changes induce modifications in the methylation status of key genes is beneficial to development of the embryos.

Results from our current study may provide a novel insight into avian evolution. Because environmental factors have an important influence on DNA methylation during early developmental stages, epigenetic markers acquired in embryonic periods could influence adult phenotypes that could be inherited in future generations via heritable DNA methylation patterns (Renaudeau et al., 2012). Evidence from our study may also have agronomic value in animal breeding, taking into account the possibility that the demands for poultry meat will rise in the near future, and breeding workers must have a comprehensive understanding regarding high quality phenotypes that can be obtained by appropriately manipulating the environmental factors in breeding conditions (Frésard et al., 2013). However, it appear limited that in present study we focus on the effect of incubation temperature on DNA methylation, the underlying relation between embryonic temperature and epigenetic modification need further investigation
